# Strain-insensitive flexible anomalous Hall-effect sensors for interactive wearables

**DOI:** 10.1038/s44306-026-00149-9

**Published:** 2026-05-07

**Authors:** Rui Xu, Eduardo Sergio Oliveros-Mata, Gilbert Santiago Canon Bermudez, Tobias Kosub, Minjeong Ha, Emily E. Evans, Jessica A.-C. Liu, Joseph B. Tracy, Denys Makarov

**Affiliations:** 1 Institute of Ion Beam Physics and Materials Research, Helmholtz-Zentrum Dresden-Rossendorf e.V., Dresden, Germany; 2 NaMLab gGmbH, Dresden, Germany; 3 Department of Materials Science and Engineering, Gwangju Institute of Science and Technology, Gwangju, Republic of Korea; 4 Department of Physics and Astronomy, Elon University, Elon, NC USA; 5 Department of Materials Science and Engineering, North Carolina State University, Raleigh, NC USA

**Keywords:** Engineering, Materials science, Physics

## Abstract

Anomalous Hall effect (AHE) sensors present a compelling platform for emerging interactive electronics. Practical integration in flexible electronics, however, has been limited by the large Hall signal drift occurring under mechanical deformation. In this work, we micropatterned Hall crosses with 1.5-nm-Co/1-nm-Pt bilayers on 2.5-μm-thick ultrathin foils, achieving a high sensitivity of 100 Ω T⁻¹ and stable sensing performance down to a bending radius of 200 μm. To ensure stable performance under continuous bending, we employed a zero-offset Hall (ZOH) measurement strategy to decouple mechanical deformation effects from the magnetic response. In this scheme, the current direction through the Hall cross is alternated and the corresponding signals are averaged, effectively canceling out deformation-induced artifacts in the magnetic measurements. Owing to their mechanical conformability and strain-insensitive operation, the ZOH-enabled AHE sensors demonstrate strong potential for interactive wearables, as exemplified in on-skin human-machine interfaces for Morse code communication and on-skin presenter control, as well as in magnetic soft robots with on-board sensing.

## Introduction

Electronics have been integrated into nearly every aspect of modern life. Within a broad spectrum of electronic devices, magnetic field sensors occupy an indispensable position^1–6^. Benefiting from their strong environmental adaptability and non-contact operating principles, these sensors have found widespread applications in navigation^7,8^, industrial automation^9^, medical instrumentation^10,11^, and consumer electronics^12,13^, etc. Among the various types of magnetic sensors, anomalous Hall effect (AHE) sensors stand out owing to their combination of unique advantages^14,15^. In contrast to giant magnetoresistive^16–18^ and tunneling magnetoresistive sensors^19,20^, which typically rely on complex ultrathin multilayer architectures or harsh post treatments, AHE sensors exhibit a markedly simpler device configuration and fabrication process, offering significant advantages in terms of cost and scalability. Despite their structural simplicity, AHE sensors achieve high sensitivity owing to the strong spin-orbit coupling at the heavy metal/ferromagnetic bilayer interface^21–23^. Notably, AHE sensors are inherently sensitive to magnetic fields applied perpendicular to the sensor plane, favorably positioning them compared to their conventional magnetoresistive counterparts. Such directional sensitivity is of particular importance for applications requiring precise single-axis measurements, e.g., systems necessitating accurate detection of tilt or magnetic field orientation. Although magnetoresistive spin valves can also achieve strong out-of-plane sensitivity, their fabrication involves highly sophisticated processes^24–26^. Further, the linear response characteristics of AHE sensors provide an additional advantage by facilitating simplified signal processing, thereby broadening their potential utility in practical applications.

The rapid development of wearable electronics^27–31^, smart healthcare^32–34^, virtual/augmented reality^18,35^, and soft robotics^36–40^ has significantly expanded the application scope of AHE sensors, while simultaneously placing higher requirements on their performance. To adapt to complex operating environments where continuous mechanical deformation, surface irregularities, and external perturbations are inevitable, AHE sensors must combine mechanical flexibility with stable electrical performance and robust magnetic responses, thereby meeting the stringent requirements for comfort, portability, and durability in next-generation intelligent devices.

In recent years, significant progress has been made in the development of flexible Hall sensors relying on different physical effects including planar Hall effect^41^, normal Hall effect^42–44^, AHE^45–47^. Thin-film technologies have been extensively employed to pattern conventional sensing materials on flexible substrates. For instance, micropatterned permalloy Hall crosses fabricated on polyethylene terephthalate (PET) foils have demonstrated stable operation under bending radii as small as 1 mm^41^. In parallel, two-dimensional (2D) materials have been increasingly incorporated into device architectures^44^. Notably, Kaidarova et al. reported a laser-scribing approach for the direct fabrication of graphene-based Hall sensors, which maintained functionality under bending with a radius of 2 mm^43^. This method requires only a single processing step and avoids the use of toxic chemicals, substantially reducing technological barriers to large-scale production. In addition, additive printing techniques have emerged as a promising route toward cost-effective and scalable fabrication of flexible Hall devices^47^. Despite these advances, mechanical deformation and interfacial instabilities in real-world application scenarios could cause signal fluctuations, degraded sensitivity, and, in severe cases, device failure. Consequently, ensuring long-term operational stability and reliability under continuous mechanical stress persists as a critical obstacle for the practical deployment of flexible Hall sensors.

In this work, we successfully develop flexible AHE sensors with robust functionality under continuous deformation. The sensors are based on perpendicularly magnetized Co/Pt-based stacks sputtered onto 2.5-μm-thin Mylar foils. Co/Pt-based AHE structures possess strong yet tunable perpendicular magnetic anisotropy, which is critical for achieving high-performance AHE sensors. By precisely engineering the layer thicknesses (1.5 nm Co and 1 nm Pt), a maximum sensitivity of approximately 100 Ω T⁻¹ is achieved. Owing to the ultrathin nature of both the active sensing bilayer and the substrate, the sensors exhibit extraordinary bending capability down to about 200 μm radius and thus excellent conformability to diverse surfaces, including paper, plastic tape, and human skin. To enhance operational stability upon mechanical deformations, we employed a zero-offset Hall (ZOH) measurement strategy (a.k.a. current-spinning technique), in which the current direction through the Hall cross is alternated, and the corresponding resistance readings are averaged. This approach provides pure Hall signals (transverse resistance), while relegating artifacts originating from geometrical asymmetry and mechanical deformations into a separate longitudinal resistance reading. In contrast, conventional Hall measurements conflate the actual Hall information and mechanical artifacts, making that technique inappropriate for flexed Hall sensors. The newly developed mechanical conformability and strain-insensitive performance of the AHE sensors enable a broad range of practical applications, as demonstrated in on-skin human-machine interfaces for Morse code communication and on-skin presenter control, as well as in self-sensing soft robotic systems.

## Results

### Fabrication of AHE sensors and ZOH measurement configuration

**Fig. 1:**
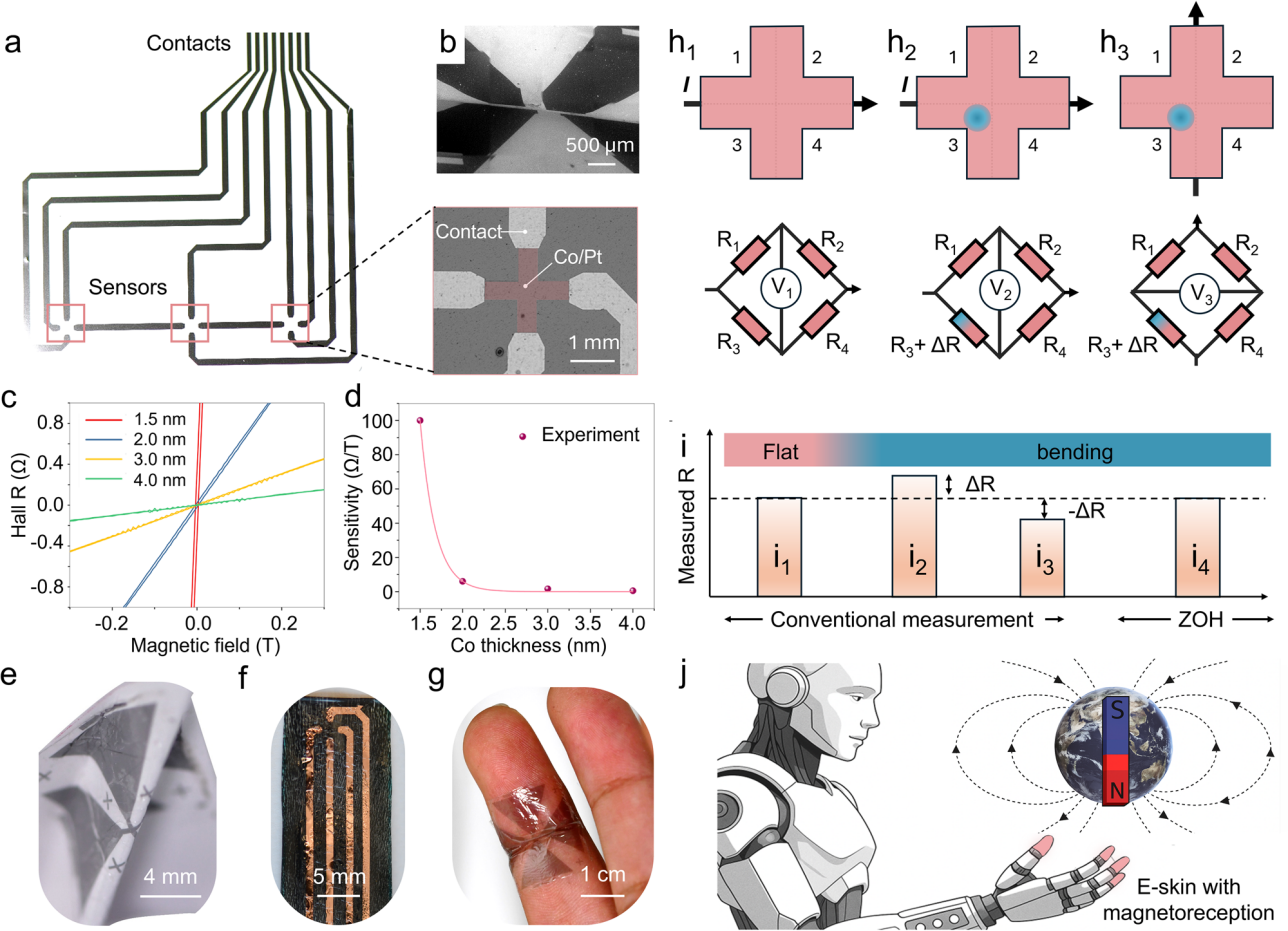
Fabrication and zero-offset measurement of flexible anomalous Hall effect (AHE) sensor. **a** Optical photograph of an AHE sensor array (left) and zoomed-in scanning electron microscopy (SEM) image of the sensing element marked in pink (right). **b** Flexible AHE sensor under mechanical bending. **c** Hall resistance and **d** sensitivity of the AHE sensors as a function of the Co layer thickness. Sensors mounted on different substrates, including **e** paper, **f** adhesive tape, and **g** skin. **h** Schematics of the AHE sensor measurement configurations. From left to right: **h**_**1**_ flat sensor without strain, characterized by the conventional measurement method; **h**_**2**_, **h**_**3**_ Deformed sensor due to applied mechanical strain (highlighted in blue), measured by conventional methods with different current directions: **h**_**2**_ left to right, **h**_**3**_ bottom to top. **i** Schematic comparison of Hall resistance measured using a four-point configuration: **i**_**1-3**_ approaches illustrated in **h**_**1-3**_ and the zero-offset Hall (ZOH) approach realized by combining configurations (**h**_**2**_, **h**_**3**_). By sequentially alternating the measurement direction and averaging the signals, the offsets in configurations (**i**_**2**_, **i**_**3**_) mutually canceled, effectively decoupling deformation effects from the Hall resistance signal in configuration (**i**_**4**_). **j** Potential application of ZOH-aided AHE sensors, exemplified by functional skin for humanoid robots.

The AHE sensors are fabricated using thin-film deposition techniques, specifically combining magnetron sputtering with standard photolithographic patterning processes. As illustrated in Fig. 1a, three AHE sensors are integrated on a substrate together with their conductive electrodes. Each sensor adopts a typical Hall-cross geometry with a channel width of 400 μm, enabling reliable transport measurements. The well-established magnetron sputtering and lithographic patterning processes ensure highly reproducible sensor performance across different fabrication batches (Fig. S1). The AHE sensor is based on a Co/Pt (1 nm) bilayer system. The Co thickness is tuned to 4, 3, 2, and 1.5 nm (Fig. 1c)^48,49^. A strong enhancement of sensitivity is observed as the Co thickness decreases, reaching a maximum sensitivity of about 100 Ω T⁻¹ (Fig. 1d). These results are consistent with prior studies^50,51^. Although further reducing the thickness, e.g., to 1 nm can significantly enhance the sensitivity to 1650 Ω T⁻¹, the pronounced hysteretic behavior renders this configuration unsuitable for sensing applications (Fig. S2). In contrast, the 1.5-nm-Co sample exhibits a near-linear response in the vicinity of zero magnetic field while remaining high sensitivity. In addition, excessively thin sensing layers are susceptible to mechanical damage under prolonged deformation. These issues would result in a drastic increase in noise and potential device failure. We further measured the voltage noise spectral density (NSD) of the AHE sensor at multiple bias currents (Fig. S3, Table S1). The measurement was carried out at room temperature without any shielding in order to evaluate sensor stability in open environment. For practical wearable operation we identify an optimal bias region (~50–110 µA) in which the voltage noise remains close to the Johnson floor (~10 nV/√Hz) while transduction increases with current; above this region detectivity deteriorates due to increased *1/f* noise. At the optimal bias of 50.4 μA, the calculated magnetic field detectivity at 1 Hz is approximately 1.92 μT/√Hz (Table S1), demonstrating that our device maintains a high signal-to-noise ratio, essential for detecting weak magnetic signatures in interactive wearable applications.

Ultrathin Mylar foils with a thickness of 2.5 μm are employed as flexible substrates, endowing the AHE sensors with remarkable mechanical conformability. The resulting devices can withstand extreme bending with radii of curvature down to several hundred micrometers without delamination (Fig. 1b). Owing to this flexibility, the sensors readily conform to a wide variety of nonplanar and dynamically deformable surfaces, including commonplace materials such as paper (Fig. 1e), adhesive tape (Fig. 1f), and even human skin (Fig. 1g). Such conformability greatly expands possible application scenarios. For example, these flexible sensors exhibit promising potentials for integration into emerging flexible technologies and adaptive electronics, where lightweight architectures and mechanical adaptability are indispensable.

However, mechanical deformation inevitably introduces signal disturbances, leading to malfunction or even complete failure of the sensor system. A Hall cross can be modeled as a Wheatstone bridge, in which an input current flows through the device and a bridge (or cross) voltage is generated perpendicular to the current direction (Fig. 1h_1_). In the ideal case, the Wheatstone bridge remains perfectly balanced and exhibits a net voltage difference of Δ*V* = 0 V, as the four resistive arms possess identical values, i.e.,

1 $$\Delta {V}_{1}=\frac{{R}_{4}{R}_{1}-{R}_{3}{R}_{2}}{{R}_{1}+{R}_{3}{+R}_{2}+{R}_{4}}=\,0$$

Upon flexing the sensors, mechanical bending or stretching induces local strains, which alter the resistance of individual arms (Fig. 1h_2,3_). These strain-induced electrical variations disturb the symmetry of the Wheatstone bridge, leading to a finite offset voltage (Δ*V* ≠ 0 V) even if no magnetic field is applied to the sensor. The deformation-induced offsets measured in the two configurations illustrated in Fig. 1h_2,3_ can be quantitatively expressed as:

2 $$\Delta {V}_{2}=\frac{{R}_{4}{R}_{1}-{{(\Delta R+R}_{3})R}_{2}}{{R}_{1}+{R}_{3}{+R}_{2}+{R}_{4}}=\,\frac{-{\Delta R\times \,R}_{2}}{{R}_{1}+{R}_{3}{+R}_{2}+{R}_{4}}$$

3 $$\Delta {V}_{3}=\frac{{{(\Delta R+R}_{3})R}_{2}-{R}_{4}{R}_{1}}{{R}_{1}+{R}_{3}{+R}_{2}+{R}_{4}}=\,\frac{{\Delta R\times R}_{2}}{{R}_{1}+{R}_{3}{+R}_{2}+{R}_{4}}$$

These equations clearly demonstrate that the imbalance in resistive elements caused by mechanical deformations is the origin of spurious voltage offsets. We note that no Hall effect had to be invoked to explain how strain-related signals occur in the bridge or cross reading, which highlights that strain-induced signal changes are longitudinal resistance effects, in stark contrast to magnetically induced Hall signals, which are transverse resistance effects. In a conventional Hall cross measurement, both phenomena will conflate in the single accessible data channel (the cross voltage or resistance), masking the contribution of the individual effects to the final reading and making inferring the magnetic field highly unreliable. By carefully compensating for the longitudinal-resistance-induced contributions to the cross reading via a ZOH measurement, the pure transverse resistance can be recovered and we can achieve

4 $$\frac{\Delta {V}_{2}+\,\Delta {V}_{3}}{2}=0$$

even for the case of mechanically flexible AHE sensors.

This concept provides a powerful strategy for suppressing parasitic mechanical effects, which can be remarkably stronger than small magnetic-field-induced signals. Such robustness is particularly critical for practical applications of flexible magnetic field sensors, where mechanical deformations are unavoidable. For example, when integrated into wearable devices or functional e-skins of humanoid robots, the ZOH-aided AHE sensor can act as a stable touchless interactive platform under dynamic mechanical bending conditions, thereby enabling seamless perception and communication with surrounding magnetic fields (Fig. 1j). Although this approach has been used to suppress offset originating from geometry imperfections and thermal gradients^52,53^, its effectiveness has not been validated for flexible applications associated with continuous deformations.

### Characterization of AHE sensors

To experimentally validate the ZOH strategy for flexible AHE sensors, a series of measurements are performed. The Hall resistance is first characterized under static bending using both the conventional Hall configuration and the ZOH approach. The conventional Hall measurement exhibits pronounced instability, with the resistance signal displaying significant deviations when the bending radius is reduced to 3.1 mm, resulting in offsets of several ohms. In contrast, the ZOH strategy effectively suppresses these deformation-induced artifacts: the Hall signal retains a linear dependence with a minimal offset confined within about ±0.1 Ω.

**Fig. 2:**
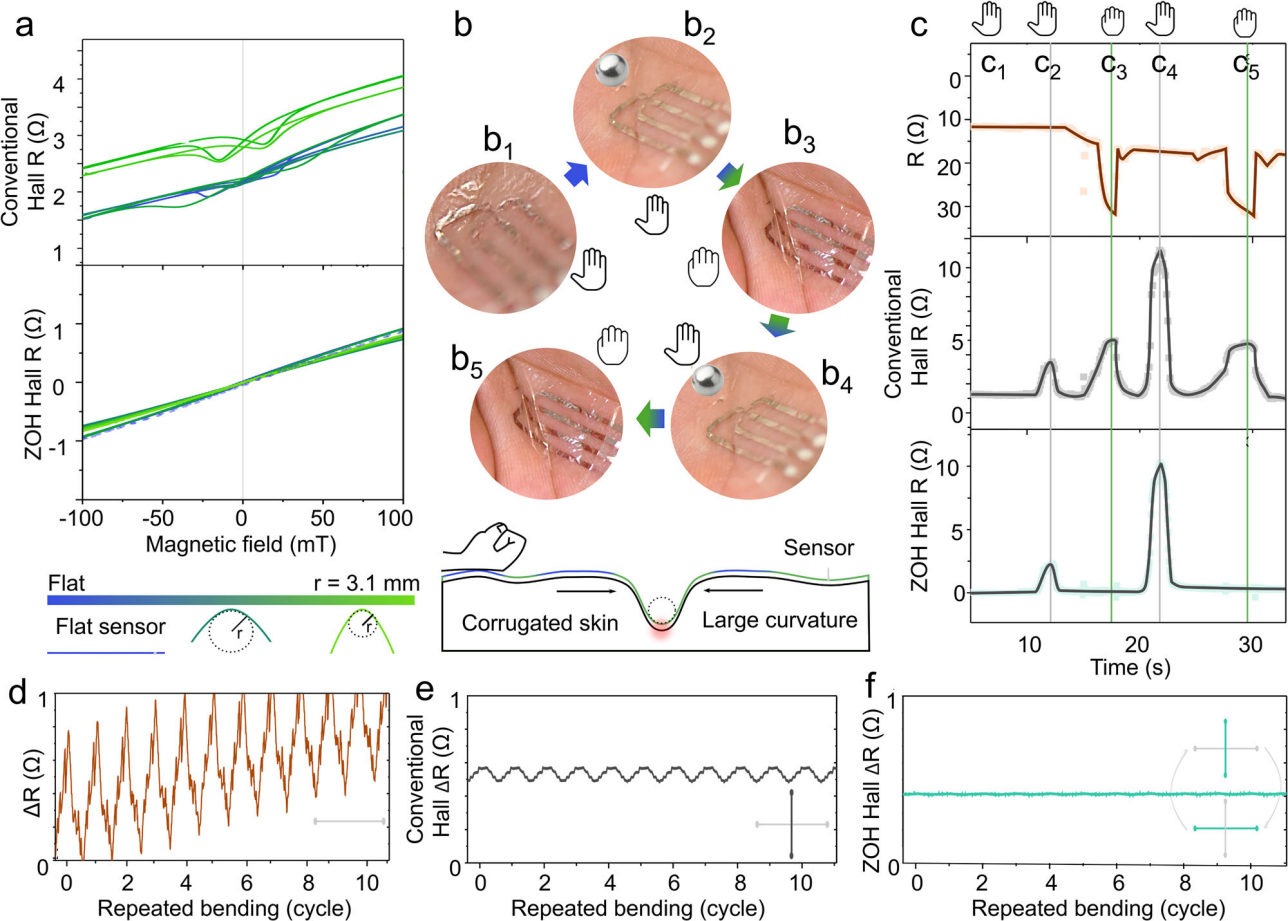
Flexible AHE sensor characterization. **a** Hall resistance of the AHE sensor as a function of magnetic field, measured using both conventional and ZOH methods. During measurement, the sensor is gradually deformed from a flat state to a bent state with a curvature radius of approximately 3.1 mm. **b** Flexible AHE sensor mounted on a palm (top) and schematic of a clenched palm causing sensor deformation (bottom). The sensor is designed to undergo five distinct states: **b**_**1**_ flat palm with unbent sensor; **b**_**2**_ unbent sensor with a magnet suspended above; **b**_**3**_ clenched palm with bent sensor; **b**_**4**_ relaxed palm with unbent sensor in the presence of a nearby magnet; and **b**_**5**_ bent sensor caused by hand clenching again. **c** Electrical signal variations corresponding to the five states in (**b**), from top to bottom: electrical resistance, conventional Hall resistance, and ZOH resistance. **d**–**f** Real-time electrical signals of the sensor generated during cyclic bending under a stable magnetic field of 50 mT, including **d** electrical resistance, **e** conventional Hall resistance, and **f** ZOH Hall resistance.

To assess the mechanical robustness of the ZOH method, cyclic bending tests are conducted over 600 cycles in a constant magnetic field of 50 mT (Fig. S4). Microscopic inspection performed after cyclic bending reveals the formation of microcracks in the Co/Pt thin film (Fig. S5). These microcracks, gradually formed during repeated bending cycles, lead to a slow increase in the overall longitudinal resistance of the Hall cross (Fig. 2d). In the conventional Hall measurement, the resistance signal reveals repeated fluctuations with a standard deviation of 28 mΩ, despite showing negligible long-term drift (Fig. 2e). When employing the ZOH strategy, the Hall signal exhibited neither drift nor fluctuations, with a substantially reduced standard deviation of 4 mΩ, representing more than a sixfold suppression relative to the conventional measurement scheme (Fig. 2f), indicative of its ability to compensate for strain-related artifacts. From an application perspective, the observed behavior highlights the importance of distinguishing between structural degradation effects and measurement artifacts. The ZOH method addresses the latter at the measurement level, while the former can be further mitigated through materials and structural optimization, such as crack-tolerant electrode designs, encapsulation layers, or the use of more ductile interlayers.

Further, the operational accuracy of the ZOH-aided AHE sensor is evaluated under practical application scenarios. Here, we emulate realistic wearable environments, where uncontrolled mechanical deformations and fluctuating magnetic signals coexist. Figure 2b illustrates five representative operational stages. Initially, when the sensor is mounted on a flat palm (Fig. 2b_1_), all resistance curves exhibit stable baselines (Fig. 2c_1_). When a magnetic ball is introduced above the sensor (Fig. 2b_2_), a distinct peak is observed in both the conventional Hall resistance and the ZOH Hall resistance signals (Fig. 2c_2_), reflecting the magnetic response. Upon removal of the magnetic stimulation and subsequent folding of the palm (Fig. 2b_3_), the corrugated skin induces pronounced variations in the electrical resistance and conventional Hall resistance, whereas the ZOH-aided measurement remains mostly unaffected, demonstrating its robustness against deformation-induced spurious signals (Fig. 2c_3_). Repeating the magnetic excitation (Fig. 2b_4_) further confirms that the ZOH-aided sensor continues to operate reliably, with the peak amplitude primarily determined by the distance and orientation of the magnet relative to the sensor. Finally, when the palm is folded again (Fig. 2b_5_), additional noise appears in both the conventional resistance and Hall resistance signals, yet the ZOH resistance consistently maintains a stable response (Fig. 2c_5_). These results highlight the reliability of the ZOH method to effectively decouple mechanical deformation effects from the magnetic response, thereby ensuring accurate and reliable operation of flexible AHE sensors under mechanically dynamic environments.

### On-skin ZOH-aided AHE sensors as a human-machine interface

**Fig. 3:**
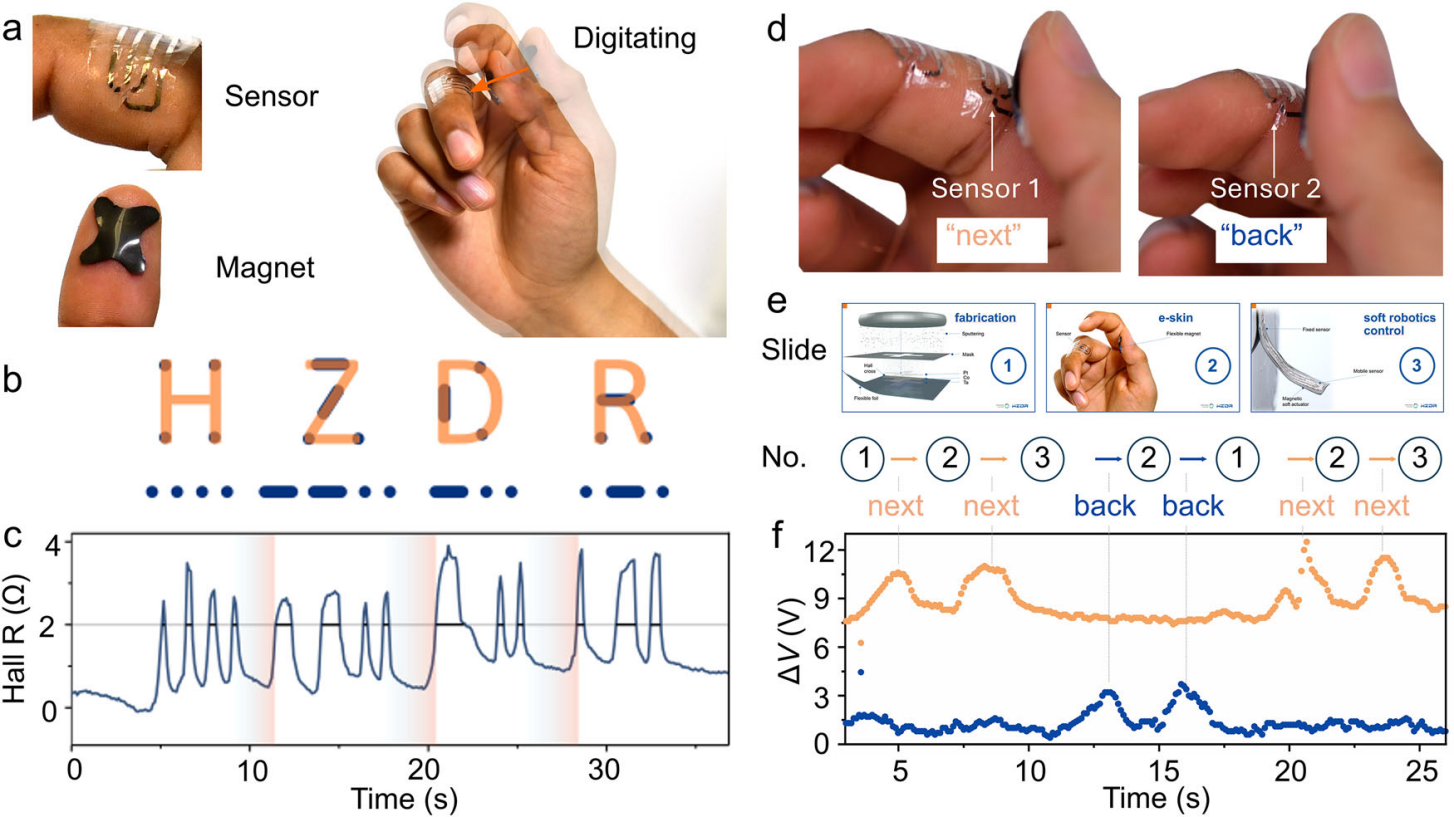
Application of flexible strain-insensitive AHE sensors. **a**–**c** On-skin Morse communication system, enabled by dynamic interaction between an AHE sensor and a magnetic resource. **a** Optical images of the AHE sensor (upper left) and magnetic skin (lower left) attached to fingers, as well as relative finger motion (right), causing magnetic field fluctuations around the sensor and thus its output signal variation. **b** Morse code information (top) and corresponding signal patterns (bottom). **c** Real-time electrical signal generated from the sensor. Variation of the sensor-magnet distance generates Hall resistance pulses, while deliberate control of the proximity time between sensor and magnet modulates the pulse width, enabling Morse code encoding. As an example, a Hall resistance trace containing four pulse groups with different widths (/short short short short/, /long long short short/, /long short short/, and /short long short/) corresponds to four letters “H”, “Z”, “D”, and “R”, i.e., the abbreviation of the corresponding author’s institute. **d**–**f** On-skin presenter based on an AHE sensor array. **d** Two sensors mounted on the forefinger, each controlling either “next slide” or “previous slide” when interacting with a magnetic skin on the thumb. **e** Demonstration of three-slide display control using the system in (**d**). **f** Output voltage responses of the two sensors to thumb-forefinger movements.

The strain-insensitive capability of the ZOH-aided AHE sensor opens promising avenues for next-generation wearable applications. As a proof-of-concept demonstration, an AHE sensor, paired with a magnetic skin composed of magnetic composite, is employed as an on-skin Morse communicator (Fig. 3a). Owing to the high sensitivity of the proposed sensors (i.e., approximately 100 Ω T⁻¹), even relatively small magnetic fields are sufficient to trigger their operation. For instance, reliable magnetoelectric responses are obtained below 15 mT, corresponding to the maximum fields generated by the magnetic composite (Figs. 3a, S6). This low-field requirement is particularly advantageous for wearable applications, as such devices necessitate continuous exposure to the human body. By rhythmically bending specific fingers, the magnetic field distribution experienced by the sensor is modulated, thereby generating a sequence of ZOH resistance signals characterized by distinguishable long and short pulses (Fig. 3c). By deliberately designing the number and temporal width of these pulses, alphanumeric information can be encoded and subsequently decoded into messages. As an illustrative example, real-time Hall resistance signals are recorded and translated into four letters “H”, “Z”, “D”, and “R” (Fig. 3b), demonstrating the feasibility of encoding linguistic information through finger motions. Please refer to supplementary Video 1 for details.

With the integration of multiple sensors, more functionalities can be realized. Here, two sensors are combined to serve as an on-skin presenter for controlling the display of a computer presentation (Fig. 3d). In this setup, sensor 1 is designated to trigger backward navigation, whereas sensor 2 is assigned to control forward navigation. For the conceptual illustration, ZOH resistance signals are recorded during the operation sequence. Specifically, two successive pulses generated by sensor 1 induced a transition of the presentation from slide 1 → slide 2 → slide 3 (Fig. 3e, f). Subsequently, two pulses generated by sensor 2 cause the display to move backward from slide 3 → slide 2 → slide 1 (Fig. 3e, f). Finally, when two pulses are again generated by sensor 1, the display advances forward once more (Fig. 3e, f). These results clearly demonstrate the feasibility of employing ZOH-aided AHE sensors to perform reliable information communication, particularly in applications where the operational environment is characterized by dynamic and continuous mechanical deformation. Notably, typical on-skin flexible electronics work in the temperature range of 20–40 °C, corresponding to ambient conditions and skin-surface temperatures. To further evaluate the device’s performance at different environments, we measured the change of the sensor response at different temperatures from 10 °C to 70 °C (Fig. S7). In this temperature range, we observe an increase in the amplitude of the AHE, which is in line with the increase of the longitudinal resistance of the metallic Co/Pt stacks with temperature. In comparison to previous flexible planar Hall effect sensors featuring either thermal robustness or strain-insensitive behavior^54,55^, the ZOH-aided AHE sensor provides a comprehensive suppression of strain-, temperature-, and resistance-induced artifacts, ensuring stable signal readout even when material-level resistance evolution is unavoidable.

### ZOH-aided AHE sensors designed for smart magnetic lifters

**Fig. 4:**
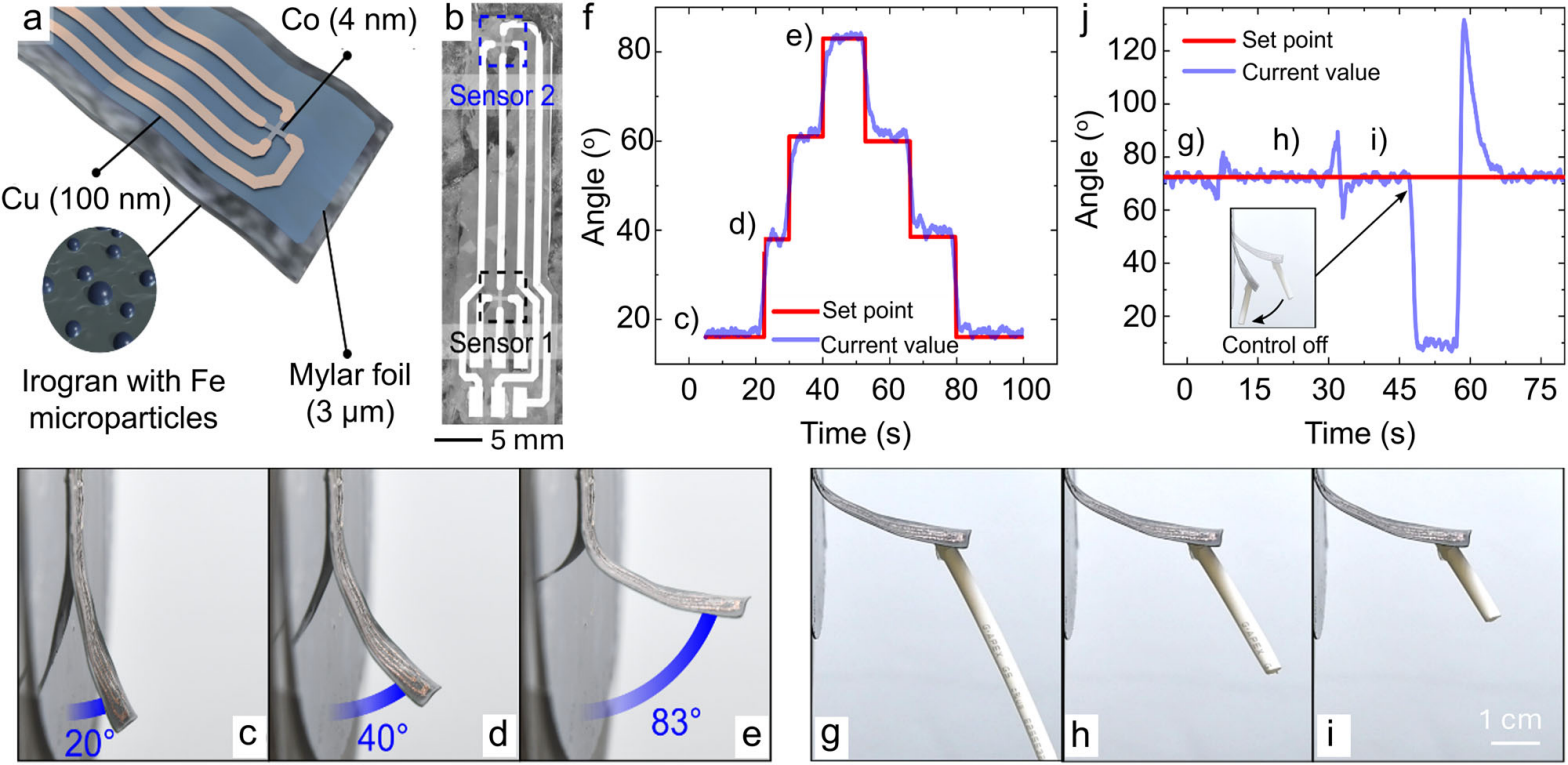
Smart magnetic lifter with motion control. **a** Schematic illustration and **b** optical micrograph of a lifter with integrated flexible AHE sensors. The magnetic soft actuator is composed of Fe microparticles dispersed in Irogran, an elastomer. Aided by a dual-sensor feedback control system, in which Sensor 1 serves as the static reference, the bending angle of the magnetic actuator integrated with Sensor 2 is calibrated based on the signal difference between the Sensor 2 and Sensor 1. **c**–**e** Sequential deformation states of the robot at different bending angles under progressively increased magnetic field strengths (left to right). **f** Correlation between the preset bending angle and the measured bending angle under magnetic actuation. **g**–**i** Actuation of the robot carrying different payloads while bending to a prescribed angle. **j** Real-time bending angle response of the smart robot to varying payloads corresponding to (**g**–**i**).

The strain-insensitive sensing capability of these sensors paves the way for advanced applications in soft robotics. Their flexible and lightweight nature allows for seamless integration with magnetic soft robots, crucially without compromising their core functionalities (Fig. 4a). The anisotropic response of the AHE sensor with the sensitivity axis strictly perpendicular to the sensor plane can be harnessed to endow soft robots with motion sensing capabilities. As an example, we integrated two sensors onto the surface of a magnetoactive elastomer soft actuator for autonomous motion control (Fig. 4b)^4,56^. A reference sensor with a fixed orientation perpendicular to the external magnetic field serves to calibrate the alignment of a second sensor that moves with the free end of the flexible cantilever (Fig. 4c–e). As the structure bends and lifts (with Sensor 2) under an external magnetic field, the ZOH resistance changes proportionally, enabling the real-time calculation of the robot’s bending angle (Fig. 4f). This capability has broad potential for precise manipulation in a range of complex environments, particularly under dynamic conditions or mechanical perturbations that could destabilize a non-sensing actuator. A compelling example is the system’s ability to maintain a stable bending amplitude when the mass loading changes (see Supplementary Video 2). Figure 4g–i shows that fixed actuation can be maintained while altering the mass that it lifts through close-loop control using the signal of the ZOH-aided AHE sensor to adjust the strength of the magnetic field of an electromagnet. In contrast, when the feedback system is deactivated, any change to the mass loading alters the bending state (Fig. 4j), underscoring the effectiveness of on-board motion sensors in providing real-time feedback that compensates for unpredictable external forces. The sensor’s ability to provide this stable feedback loop is essential for applications requiring predictable movement, such as in handling delicate objects or medical procedures.

## Discussion

In this work, we present flexible AHE sensors that exhibit strain-insensitive functionality, a critical requirement for reliable operation under mechanical deformation. These sensors achieve, to our best knowledge, the highest sensitivity among flexible Hall sensors reported to date and exceptional mechanical flexibility (Table S2). The high sensitivity is realized through systematic engineering, which involves the rational material selection (specifically, a Pt/Co-based stack with perpendicular magnetic anisotropy) and the precise thickness optimization. Benefiting from its ultrathin architecture and lightweight characteristics, the sensor can intimately conform to substrates with non-planar geometries and dynamically deforming surfaces, without compromising its electrical or magnetic performance. Further, we introduce a ZOH measurement strategy that effectively decouples strain-induced signal changes from the desired magnetic signal, thereby ensuring stable and accurate magnetic field sensing. The combination of conformal integration, mechanical robustness, and strain-insensitive operation substantially broadens the application scope of the proposed flexible AHE sensors. In particular, these features enable consistent performance in wearable electronics, where intimate contact and continuous motion are inevitable, as well as in soft robotics, where devices must maintain sensing stability under repeated and complex deformations.

Despite the successful realization of strain-insensitive functionality in this work, the practical deployment of magnetic skins as electronic skin remains confronted with substantial challenges. In real-world applications, the human finger undergoes complex, large-amplitude deformations and continuous three-dimensional reorientations, which demand not only intimate, durable skin-sensor contact but also reliable discrimination between intentional magnetic stimuli and unintended mechanical perturbations. Moreover, environmental and physiological factors such as sweat and temperature variations can induce baseline resistance drifts, placing stringent requirements on material stability, encapsulation, and signal robustness. These considerations highlight that, while the present strategy represents an important step toward mechanically decoupled magnetic sensing, significant multidisciplinary efforts are still required to achieve fully reliable and long-term wearable magnetic e-skin systems.

## Methods

### Fabrication of flexible Hall crosses

Flexible AHE devices were fabricated on 2.5 µm-thick Mylar foils (Chemplex Industries Inc., USA) laminated onto temporary 5 cm × 5 cm glass carriers using a PDMS layer (Sylgard 184, Dow Corning) spin-coated at 1000 rpm and baked for 2 h at 70 °C to facilitate handling. Hall crosses (2 mm × 2 mm with 400 µm line width) were patterned using AZ 5214E photoresist (MicroChemicals GmbH, Germany): the resist was spin-coated at 4000 rpm, soft-baked for 5 min at 90 °C, exposed using a DWL 66 laser writer (Heidelberg Instruments, Germany), post-exposure baked at 120 °C for 2 min, flood-exposed for 30 s (proMa 140 017, Germany), and developed in AZ 351B for 60 s, followed by a deionized-water rinse and compressed-air drying. Co(*x* nm)/Pt(1 nm) bilayers were then deposited by magnetron sputtering at room temperature onto the supported Mylar (argon pressure 10^−3^ mbar; base pressure 10^−7^ mbar; deposition rate 0.2 nm/s), with Co thickness *x* = {4, 3, 2, 1.5} nm. At the end, sensors were defined by lift-off in acetone without ultrasound. Electrical contacts and interconnects were defined in a second lithography step as described above and using magnetron sputtering of Ta(2 nm)/Cu(100 nm)/Pt(2 nm) stacks. Due to discrepancies in fabrication conditions (e.g., slight compositional deviations in sputtering targets, vacuum level of the chamber, substrate roughness, and contamination), variations in device performance may occur across different batches.

### Electrical and magnetic measurements

Measurements were performed at ambient conditions using a Tensormeter measurement device (HZDR Innovation GmbH, Germany) with sensor currents between 1 µA and 10 mA, modulated as a 1775 Hz sine wave. A laboratory electromagnet provided a homogeneous out-of-plane magnetic field up to 450 mT. Hall resistance was measured with and without the ZOH preset of the Tensormeter device. In ZOH mode, the Tensormeter alternates the current injection along orthogonal axes of the Hall cross, computing the longitudinal and transverse resistance tensor components. We used switching frequencies up to 20 Hz. The temperature dependence of the AHE was measured using the ZOH preset by positioning a flexible sensor on a Peltier element located between pole shoes of an electromagnet.

### Bending tests

Static bending employed a custom setup where sensors were clamped at both ends and the curvature radius was adjusted by decreasing the clamp spacing. To achieve controllable and reproducible mechanical bending, the sensor fabricated on the ultrathin Mylar substrate was laminated onto a thicker PET foil. During bending, the PET foil serves as a mechanical support layer whose thickness and elastic modulus are significantly larger than those of the Mylar substrate. To further enhance the interfacial adhesion between the Mylar foil and the PET support, an ultrathin PDMS interlayer was spin-coated onto the PET foil prior to lamination. PDMS promotes intimate conformal contact at the interfaces, ensuring that the PDMS/PET/Mylar trilayer deforms cooperatively during bending, effectively constraining the ultrathin Mylar substrate to follow the curvature imposed by the PET layer and thereby ensuring bending uniformity and mechanical stability. The local radius along the sample was extracted from optical side-view photographs. The Hall effect signal was measured in both bent and flattened states. Dynamic bending was performed using a Tensile Sample Holder of a Phenom XL SEM, cycling the sample 600 times between bending radii of 30 mm and 15 mm while recording electrical signals.

### On-skin demonstrators

A thumb-mounted magnetic skin was fabricated from NdFeB particles dispersed in PDMS (70 wt%), spin coated into a 0.3 mm-thick 8 mm × 8 mm patch and magnetized out of plane using a 2.4 T electromagnet. At separation distance for these permanent magnetic patches from 20 mm down to 2 mm, the strength of the magnetic field at the sensor location was approximately 0.5–10 mT. For on-skin placement, a 10% aqueous poly(vinyl alcohol) (PVA) adhesive was used. The skin was washed with soap, cleaned with isopropyl alcohol, and fully dried before mounting. No additional encapsulation was applied to the sensors.

For the Morse demo, a single sensor was placed on the lateral side of the middle phalanx of the middle finger, while the magnetic skin was mounted on the distal phalanx of the thumb. User generated dots by brief taps and dashes by longer presses of the thumb magnet against the sensor finger, producing pulses with target widths of 150–250 ms and 400–600 ms, separated by 150–300 ms gaps. The index finger served as a physical spacer between the thumb and sensor during tapping. Using this protocol, sequences encoding the English alphabet were recorded using a custom Labview program.

For the presenter demo, two sensors were mounted on the lateral sides of the index finger at the distal and middle phalanges, separated by approximately 20 mm, while the magnetic skin was also placed on the distal phalanx of the thumb. For slide navigation, the two index-finger sensors were mapped as follows: Sensor 1 = next slide; Sensor 2 = previous slide. Event detection used a simple threshold on the filtered signal with an amplitude criterion of approximately 2 mV above baseline and a minimum pulse width of 120–200 ms. A custom LabVIEW program performed real-time detection and emulated keyboard inputs to control the presentation. Unless otherwise stated, data were acquired with an integration time of 50 ms and a 5-point moving average.

### Soft robotic lifter control demonstration

A magnetic soft actuator was fabricated by casting a composite of Fe microparticles dispersed in Irogran, a thermoplastic polyurethane elastomer, into a PTFE mold with in-plane dimensions of 70 mm × 20 mm and a target thickness of 250 µm. The composite comprised 10 wt% Fe microparticles (Jilin Jien Nickel Industry, JCF2-2; mean diameter 4.2 µm) dispersed in Irogran (Huntsman PS 455-203). The Irogran was dissolved in tetrahydrofuran (THF), followed by addition of Fe microparticles. The mixture was vigorously stirred and vortexed, cast into the mold, and subjected to controlled solvent evaporation under an applied magnetic field of approximately 10 mT for 1 h at room temperature. The field during evaporation induced in-plane chaining and orientation of the particles, yielding a cantilever geometry optimized for field-driven bending^57,58^.

Two flexible AHE sensors were integrated on the cantilever with a center-to-center separation of 30 mm. Sensor 1 served as a reference with a fixed orientation relative to the external field, while Sensor 2 was mounted on the bending segment such that its normal rotated with the cantilever during actuation. Sensors were attached using the same water-soluble 10% PVA adhesive described for the on-skin demos and for encapsulation. The resulting signal difference between the sensors during actuation was correlated with measured angles and fit with a linear model over the tested range to obtain the angle to signal transfer function.

A laboratory electromagnet provided a homogeneous field for DC/quasi-static actuation up to 100 mT. The field was applied out of plane with respect to the reference sensor plane and the cantilever bending angles were commanded by stepping the field amplitude. In operation, Sensor 1 acted as the reference channel and Sensor 2 as the angle measurement channel. A closed-loop controller adjusted the electromagnet field to track a target bending angle using the calibrated transfer function. To evaluate robustness, different payloads were attached near the cantilever tip, then sequentially reduced by cutting with nonmagnetic scissors.

### Ethics

We applied a permanent magnet to a finger of a user in several studies reported in this manuscript. This magnet is not an electronic component. Furthermore, in several studies, a sensor is applied on skin of a user. These studies are done accordingly to the ethic approval #SR-EK-459122024 from the ethics committee at the Technical University of Dresden. For this case, we have a written consent of the user (one volunteer, male, 30 years old), who was wearing this sensor. The current lines are always isolated and are not in touch with the skin. The sensor is not worn on skin for any extended duration.

## Supplementary information

supporting information-rev3

video1

video2

## Data Availability

Data is provided within the manuscript or supplementary information files. All of the data supporting the conclusions are available within the article and the Supplementary Information. Additional data are available from the corresponding authors upon reasonable request.
