# Supplementary material for: Strain-insensitive flexible anomalous Hall-effect sensors for interactive wearables
Source: Npj Spintron. 2026 May 7;4(1):27. doi: 10.1038/s44306-026-00149-9 (PMC13331739; doi:10.1038/s44306-026-00149-9)
Supplement: Supplementary file 1 — supporting information-rev3 [file 44306_2026_149_MOESM1_ESM.pdf]

Supplementary information

Strain-insensitive flexible anomalous Hall-effect sensors for interactive wearables

Authors: *Rui Xu[1,#,]*, Eduardo Sergio Oliveros-Mata[1,2,#], Gilbert Santiago Canon Bermudez[1], Tobias Kosub[1], Minjeong Ha[1,3], Emily E. Evans[4], Jessica A.-C. Liu[5], Joseph B. Tracy[5], Denys Makarov[1]**

The supplementary information includes two videos, seven figures, and two tables.

Captions for Supplementary movies

Supplementary movie 1: On-skin Morse communication system.

Flexible AHE sensor is placed on the lateral side of the middle phalanx of the middle finger, while the magnetic skin (i.e., conformal permanent magnet patch) was mounted on the distal phalanx of the thumb. User generated dots by brief taps and dashes by longer presses of the thumb against the middle finger, producing pulses with target widths. The index finger served as a physical spacer between the magnet and sensor during tapping. Using this protocol, sequences encoding the English alphabet were recorded as exemplified with the word “HZDR”.

Supplementary movie 2: Magnetic soft actuator with on-board AHE sensor.

Flexible AHE sensors are laminated on a magnetic soft actuator. Sensor 1 served as a reference with a fixed orientation relative to the external field, while Sensor 2 was mounted on the bending segment such that its normal rotated with the cantilever during actuation. In operation, Sensor 1 acted as the reference channel and Sensor 2 as the angle measurement channel. A closed-loop controller adjusted the electromagnet field to track a target bending angle using the calibrated transfer function. To evaluate robustness, different payloads were attached near the cantilever tip, then sequentially reduced by cutting with nonmagnetic scissors.

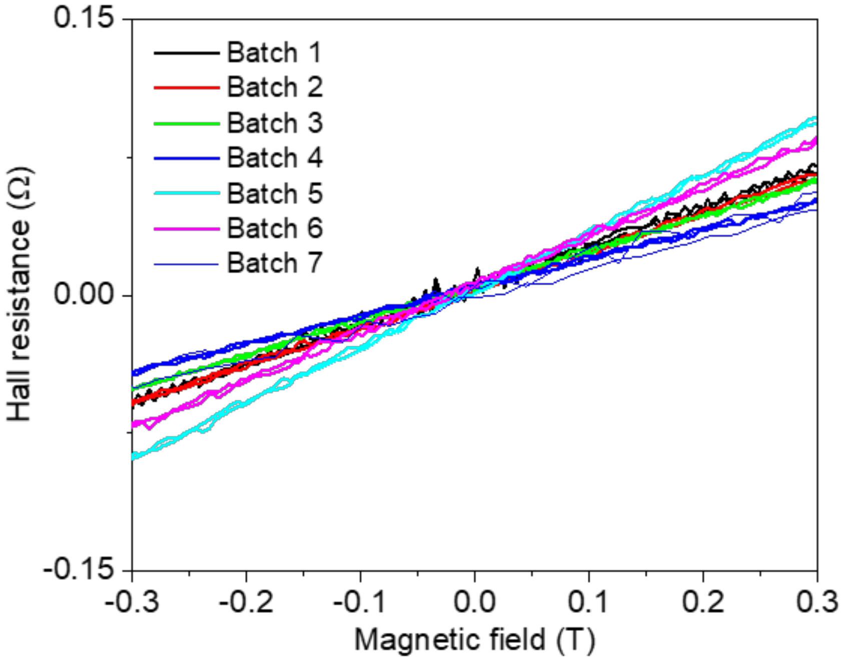

Figure S1. Comparison of the Hall resistance of AHE sensors fabricated across seven batches. The sensors exhibit comparable Hall resistance values, demonstrating high reproducibility among different fabrication batches.

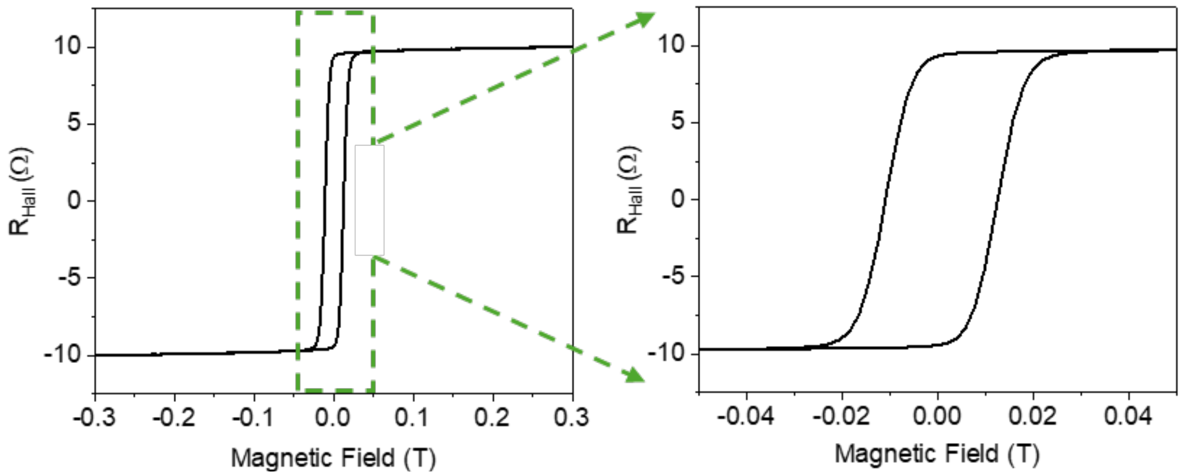

Figure S2. Hall resistance of AHE sensor with 1-nm-thick Co layer. Reducing the Co layer thickness to 1 nm can significantly enhance the sensitivity to 1650 Ω T^{-1}, however, the pronounced hysteretic behavior renders this configuration unsuitable for sensing applications.

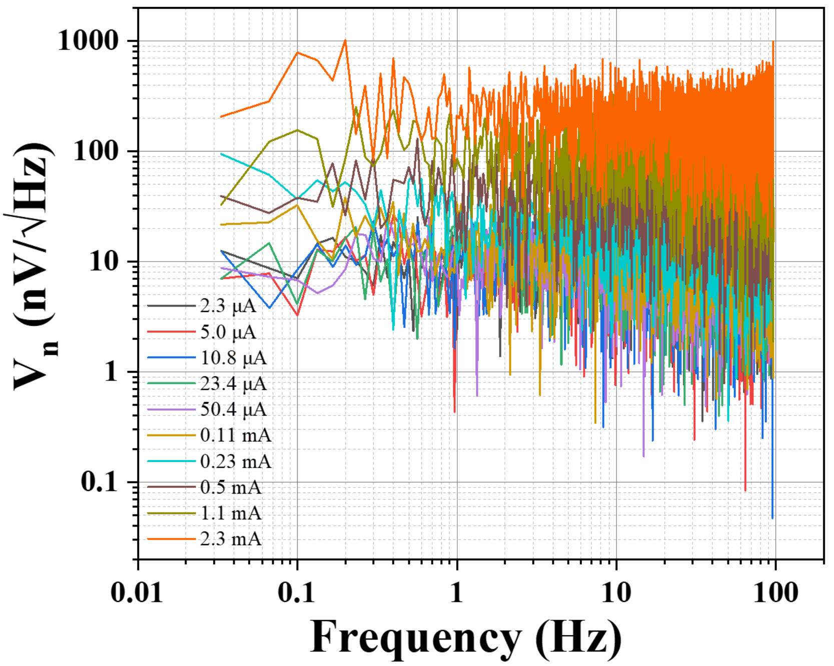

Figure S3. Voltage noise spectral density (NSD) V_n of AHE sensor measured at multiple bias currents. We measured the voltage noise spectral density (NSD) V_n of the AHE sensor at multiple bias currents from 2.3 µA to 2.3 mA (Figure S1, Table S1). The measurement was carried out at room temperature without any shielding in order to evaluate sensor stability in open environment. At room temperature, the low-bias noise floor is ~ 9 - 10 nV/√Hz, consistent with the Johnson thermal noise expected for the system and device resistance (~2438 Ω) combined with the preamplifier input noise. As the bias current increases above ~ 0.1-0.2 mA, a pronounced *1/f* component appears and the low-frequency corner frequency shifts toward higher frequencies; at the largest biases the *1/f* amplitude increases by more than an order of magnitude. This behaviour is consistent with published observations that magnetic *1/f* noise in AHE/xMR sensors grows with current density while Johnson noise remains unchanged[1,2]. The current-dependent noise is attributed to a combination of Joule heating (I^2R) that raises local temperature and enhances magnetization fluctuations, and to current-induced magnetization instabilities in ultrathin ferromagnetic layers. For practical wearable operation we identify an optimal bias region (~ 50 - 110 µA) in which the voltage noise remains close to the Johnson floor while transduction increases with current; above this region detectivity deteriorates due to increased *1/f* noise.

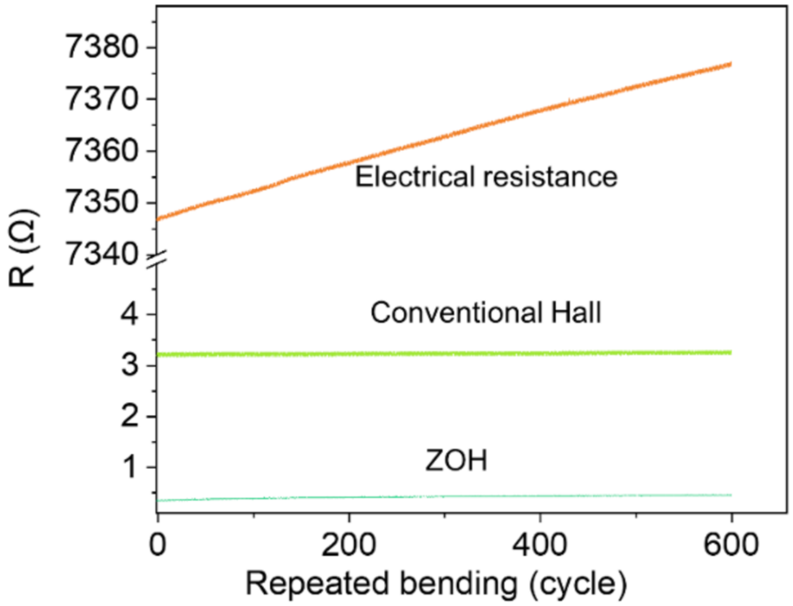

Figure S4. Electromechanical stability of the AHE sensor under cyclic bending. Temporal evolution of the sensor signals (including electrical resistance, conventional Hall and ZOH signals) recorded under a constant out-of-plane magnetic field of 50 mT during repetitive bending cycles. Although the sensor resistance exhibits drift due to cracks generated, the Hall signal remains stable. Moreover, the zero-offset Hall (ZOH) configuration effectively suppresses parasitic offset voltages, resulting in an offset much closer to zero compared with that of conventional Hall devices.

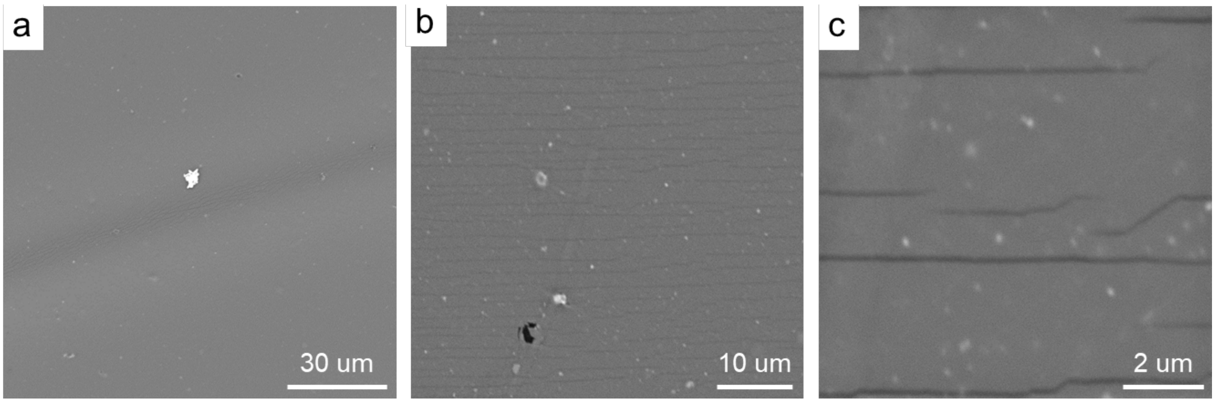

Figure S5. Representative scanning electron microscopy (SEM) micrographs of the Co/Pt sensing layer after 600 bending cycles. The images, a-c) captured at various magnifications, reveal the generation of microcracks within the active layer. These structural defects are identified as the primary source of the observed slow drift in electrical resistance during repeated mechanical deformation.

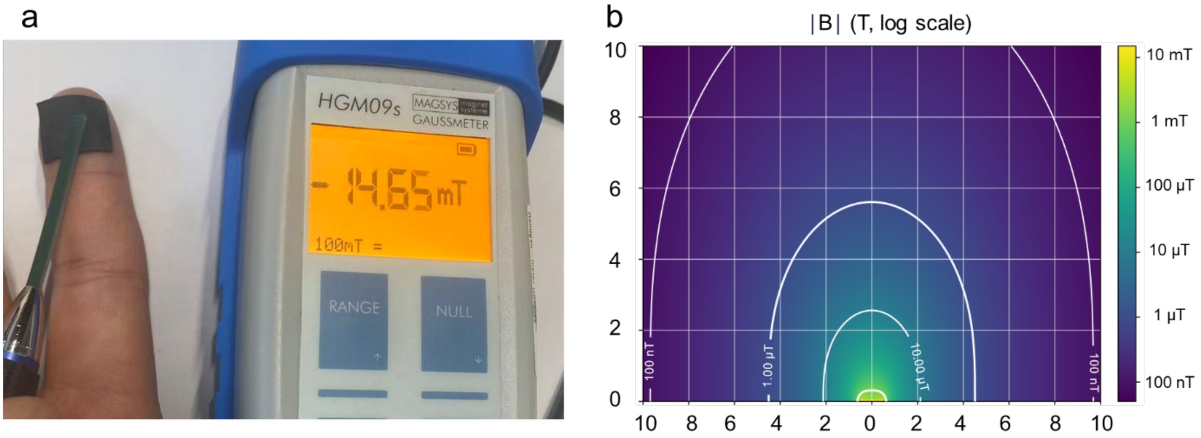

Figure S6. Magnetic fields generated by the magnetic composite-based functional skin. (a) Magnetic field measured at a point on the top surface of the composite. (b) Two-dimensional magnetic field distribution around the composite.

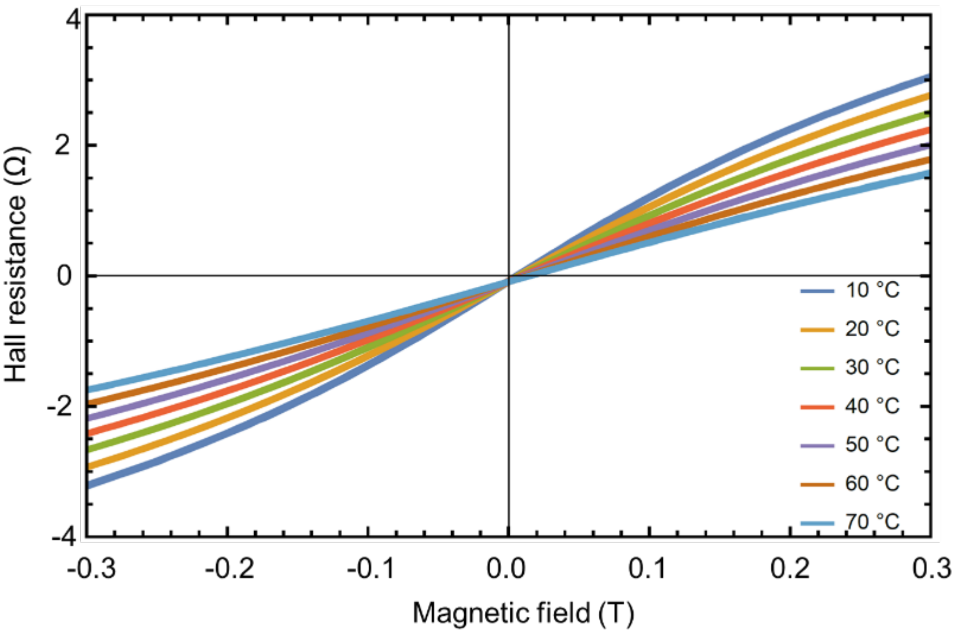

Figure S7. Change of the AHE effect with temperature.

Table S1. Noise floor of AHE sensor measured at multiple bias currents.

**Current**	**V_n (nV/√Hz) at 1 Hz**	**Detectivity (µT/√Hz) at 1 Hz**	**V_n (nV/√Hz) at 10 Hz**	**Detectivity (µT/√Hz) at 10 Hz**
2.3 µA	9.9	43.04	9.9	43.04
5.0 µA	9.5	19	9.5	19
10.8 µA	9.4	8.7	9.4	8.7
23.4 µA	10.1	4.32	10.1	4.32
50.4 µA	9.7	1.92	9.7	1.92
0.11 mA	12.1	1.1	12.1	1.1
0.23 mA	19.5	0.85	19.5	0.85
0.5 mA	39.5	0.79	39.5	0.79
1.1 mA	80.7	0.73	80.7	0.73
2.3 mA	191.9	0.83	180.2	0.78

Although Detectivity can be slightly further minimized at higher currents, 50.4 µA is regarded as the optimal trade-off point. This selection accounts for the stringent power constraints of wearable electronics and avoids excessive Joule heating that could compromise device reliability

Table S2. Comparison of flexible magnetic field sensors

Sensor type	Substrate	Sensing material system	Sensitivity	Bending radius	Ref.
Hall	Kapton	Graphene	~1.12 V/AT	5 mm	3
Hall	Kapton	Graphene	79 V/AT	5 mm	4
Hall	Polyimide/polyether ether ketone	Bismuth	2.3 V/AT	6 mm	5
Hall	Polyether ether ketone	Bismuth	2.3 V/AT	5 mm	6
AMR	Polyethylene terephthalate	Permalloy (Py)	5 V/T	5 mm	7
AMR	Mylar foil	Py	190% / T	20 µm	8
AMR	Polyethylene terephthalate	Py	0.86 V/T	1 mm	9
AMR	Polydimethylsiloxane	NiFe/IrMn	0.74 µV/Oe	-	10
GMR	Polyester	Co/Cu multilayers	-	22 mm	11
GMR	Polydimethylsiloxane	Co/Cu multilayers	-	-	12
GMR	Polydimethylsiloxane	Py/Cu multilayers	220%/T	-	13
GMR	Polyethylene terephthalate	Co/Cu multilayers	220%/T	3 µm	14
GMR	Flexible FPC board	Co/Cu multilayers	93%/T	22 mm	15
Spin valve	Polydimethylsiloxane	Ta/IrMn/CoFe/Cu/CoFe/Py	0.2% /Oe	-	16
Spin valve	Polydimethylsiloxane	CoFe/Cu/CoFe /IrMn/Pt/Ta	0.2% /Oe	-	17
AHE	2.5 µm-thick Mylar foils	Co/Pt	100 Ω/T	200 µm	This work

It should be noted that the figures of merit for magnetic field sensors vary depending on the sensing mechanism. While this complicates a direct numerical benchmarking, Table S2 provides an overview of the state-of-the-art performance of the sensors as reported in the original publications.

References:

1. Monteblanco, E. *et al.* Constant TMR magnetic field sensor detectivity with bias voltage. *arXiv:2103.04750* 25–30 (2021) doi:10.48550/arXiv.2103.04750.

2. Nguyen, T. N. A., Do, Q. N. P. K. T., Pham, H. K. V. H. N. & Do, D. T. T. H. M. The study of the effect of device downsizing on 1/f noise in deep submicron magnetic tunnel junctions. *Appl. Phys. A* **131**, 505 (2025).

3. Kaidarova, B. A. *et al.* Flexible Hall sensor made of laser-scribed graphene. *npj Flex. Electron.* **5**, 2 (2021).

4. Wang, Z., Shaygan, M., Otto, M., Schall, D. & Neumaier, D. Flexible Hall sensors based on graphene. *Nanoscale* **8**, 7683–7687 (2016).

5. Melzer, M. *et al.* Wearable magnetic field sensors for flexible electronics. *Adv. Mater.* **27**, 1274–1280 (2015).

6. Mönch, I. J. *et al.* Flexible Hall Sensorics for Flux-Based Control of Magnetic Levitation. **51**, 4004004 (2015).

7. Wang, Z. *et al.* Highly Sensitive Flexible Magnetic Sensor Based on Anisotropic Magnetoresistance Effect. *Adv. Mater.* **28**, 9370–9377 (2016).

8. Oliveros Mata, E. S. *et al.* Printable anisotropic magnetoresistance sensors for highly compliant electronics. *Appl. Phys. A Mater. Sci. Process.* **127**, 280 (2021).

9. Granell, P. N. *et al.* Highly compliant planar Hall effect sensor with sub 200 nT sensitivity. *npj Flex. Electron.* **3**, 3 (2019).

10. Ozer, B., Piskin, H. & Akdogan, N. Shapeable Planar Hall Sensor With a Stable Sensitivity Under Concave and Convex Bending. **19**, 5493–5498 (2019).

11. Chen, Y. F. *et al.* Towards flexible magnetoelectronics: Buffer-enhanced and mechanically tunable GMR of Co/Cu multilayers on plastic substrates. *Adv. Mater.* **20**, 3224–3228 (2008).

12. Melzer, M. *et al.* Stretchable magnetoelectronics. *Nano Lett.* **11**, 2522–2526 (2011).

13. Melzer, M. *et al.* Direct transfer of magnetic sensor devices to elastomeric supports for stretchable electronics. *Adv. Mater.* **27**, 1333–1338 (2015).

14. Melzer, M. *et al.* Imperceptible magnetoelectronics. *Nat. Commun.* **6**, 6080 (2015).

15. Karnaushenko, D. *et al.* High-performance magnetic sensorics for printable and flexible electronics. *Adv. Mater.* **27**, 880–885 (2015).

16. Melzer, M., Lin, G., Makarov, D. & Schmidt, O. G. Stretchable spin valves on elastomer membranes by predetermined periodic fracture and random wrinkling. *Adv. Mater.* **24**, 6468–6472 (2012).

17. Li, H. *et al.* Stretchable Spin Valve with Stable Magnetic Field Sensitivity by Ribbon-Patterned Periodic Wrinkles. *ACS Nano* **10**, 4403–4409 (2016).
